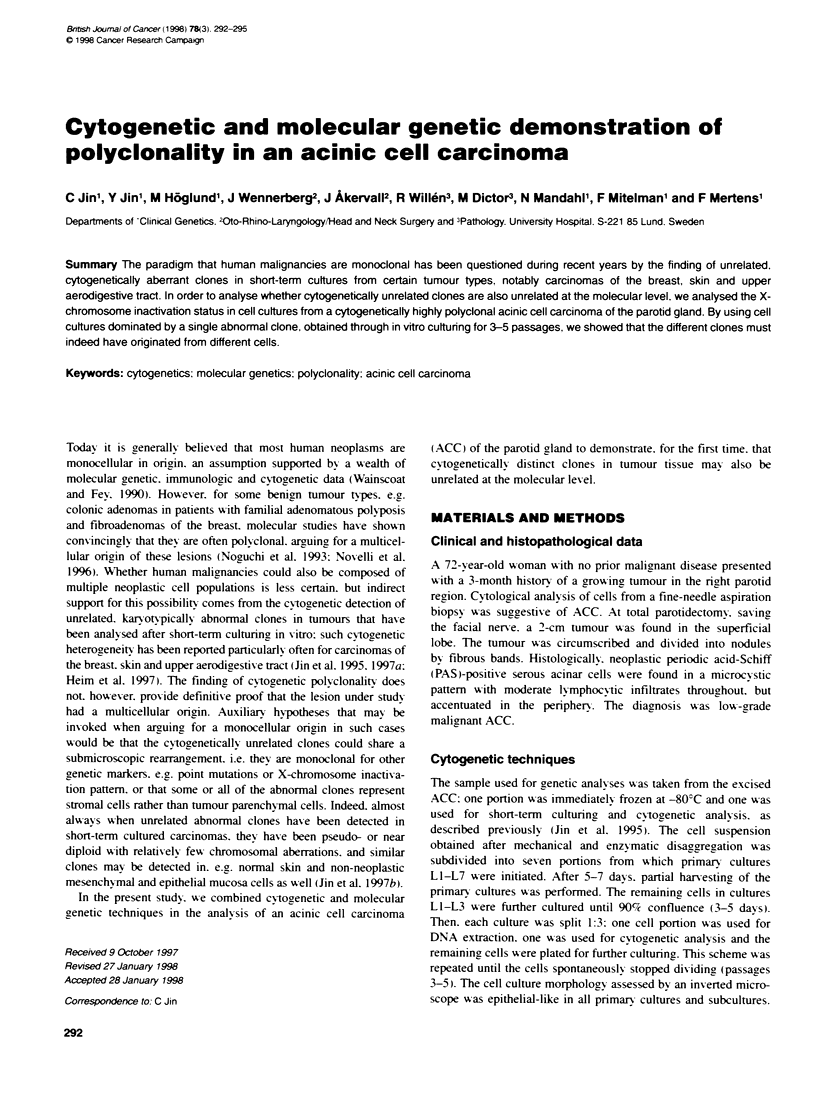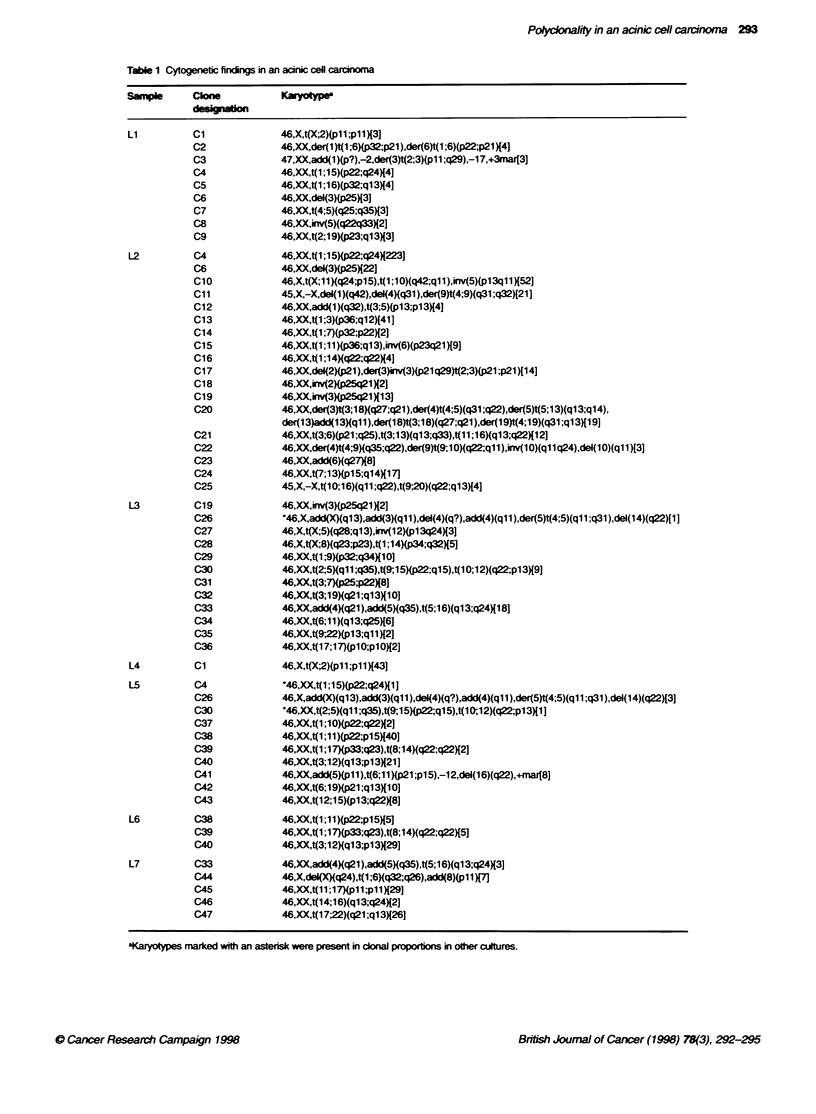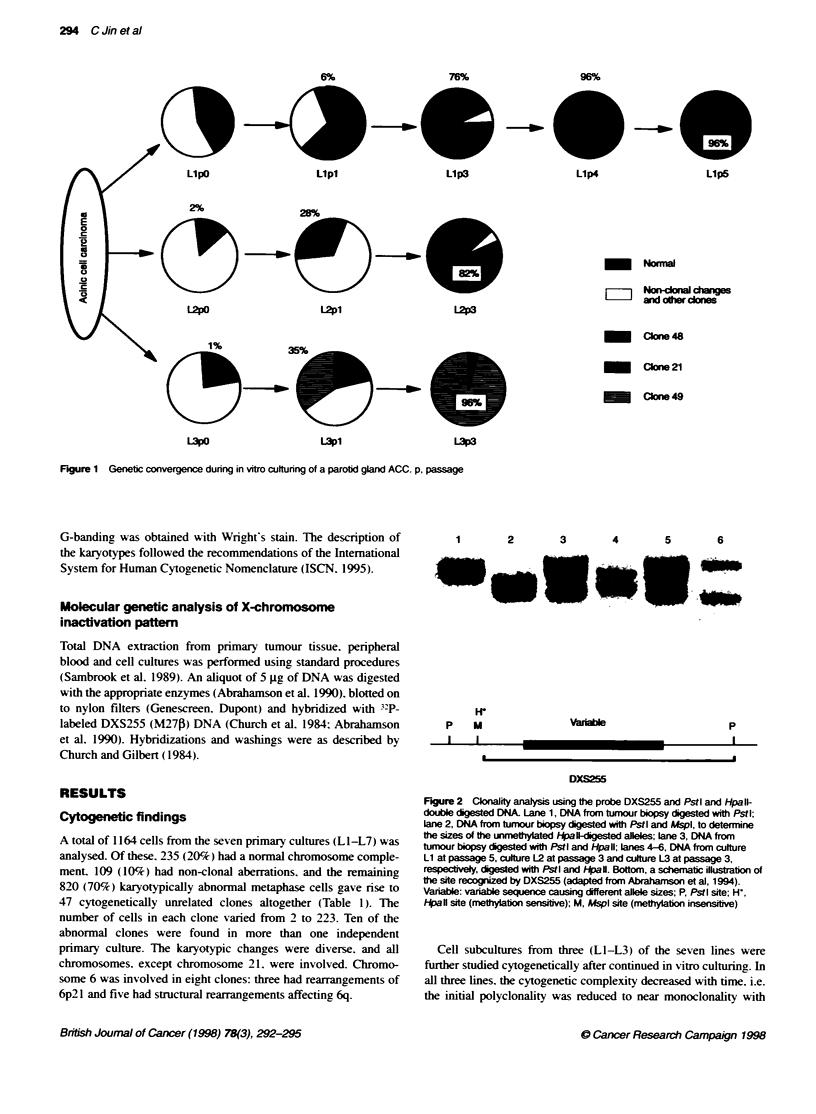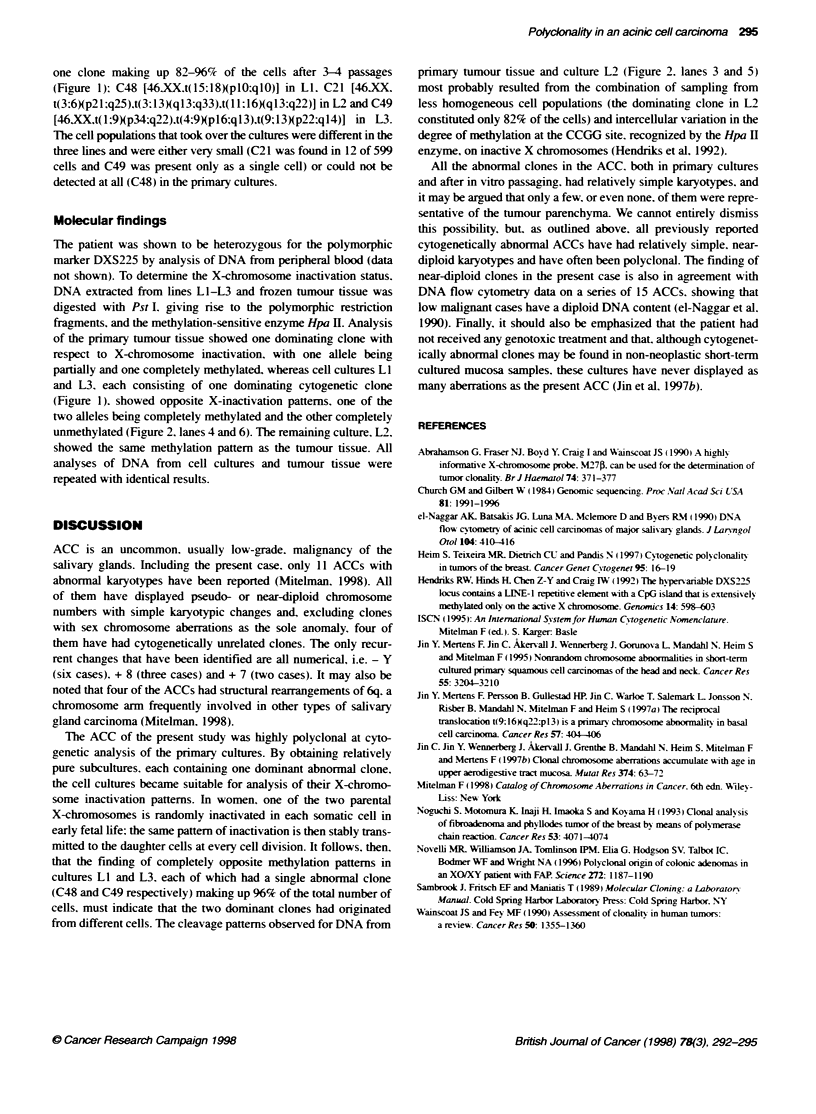# Cytogenetic and molecular genetic demonstration of polyclonality in an acinic cell carcinoma.

**DOI:** 10.1038/bjc.1998.489

**Published:** 1998-08

**Authors:** C. Jin, Y. Jin, M. Höglund, J. Wennerberg, J. Akervall, R. Willén, M. Dictor, N. Mandahl, F. Mitelman, F. Mertens

**Affiliations:** Department of Clinical Genetics, University Hospital, Lund, Sweden.

## Abstract

**Images:**


British Journal of Cancer (1998) 78(3), 292–295
© 1998 Cancer Research Campaign

### Cytogenetic and molecular genetic demonstration of polyclonality in an acinic cell carcinoma

C Jin[1], Y Jin[1], M Höglund[1], J Wennerberg[2], J Åkervall[2], R Willén[3], M Dictor[3], N Mandahl[1], F Mitelman[1] and F Mertens[1]

Departments of [1]Clinical Genetics, [2]Oto-Rhino-Laryngology/Head and Neck Surgery and [3]Pathology, University Hospital, S-221 85 Lund, Sweden

**Summary** The paradigm that human malignancies are monoclonal has been questioned during recent years by the finding of unrelated, cytogenetically aberrant clones in short-term cultures from certain tumour types, notably carcinomas of the breast, skin and upper aerodigestive tract. In order to analyse whether cytogenetically unrelated clones are also unrelated at the molecular level, we analysed the X-chromosome inactivation status in cell cultures from a cytogenetically highly polyclonal acinic cell carcinoma of the parotid gland. By using cell cultures dominated by a single abnormal clone, obtained through in vitro culturing for 3–5 passages, we showed that the different clones must indeed have originated from different cells.

**Keywords:** cytogenetics; molecular genetics; polyclonality; acinic cell carcinoma

Today it is generally believed that most human neoplasms are monocellular in origin, an assumption supported by a wealth of molecular genetic, immunologic and cytogenetic data (Wainscoat and Fey, 1990). However, for some benign tumour types, e.g. colonic adenomas in patients with familial adenomatous polyposis and fibroadenomas of the breast, molecular studies have shown convincingly that they are often polyclonal, arguing for a multicellular origin of these lesions (Noguchi et al, 1993; Novelli et al, 1996). Whether human malignancies could also be composed of multiple neoplastic cell populations is less certain, but indirect support for this possibility comes from the cytogenetic detection of unrelated, karyotypically abnormal clones in tumours that have been analysed after short-term culturing in vitro; such cytogenetic heterogeneity has been reported particularly often for carcinomas of the breast, skin and upper aerodigestive tract (Jin et al, 1995, 1997*a*; Heim et al, 1997). The finding of cytogenetic polyclonality does not, however, provide definitive proof that the lesion under study had a multicellular origin. Auxiliary hypotheses that may be invoked when arguing for a monocellular origin in such cases would be that the cytogenetically unrelated clones could share a submicroscopic rearrangement, i.e. they are monoclonal for other genetic markers, e.g. point mutations or X-chromosome inactivation pattern, or that some or all of the abnormal clones represent stromal cells rather than tumour parenchymal cells. Indeed, almost always when unrelated abnormal clones have been detected in short-term cultured carcinomas, they have been pseudo- or near diploid with relatively few chromosomal aberrations, and similar clones may be detected in, e.g. normal skin and non-neoplastic mesenchymal and epithelial mucosa cells as well (Jin et al, 1997*b*).

In the present study, we combined cytogenetic and molecular genetic techniques in the analysis of an acinic cell carcinoma (ACC) of the parotid gland to demonstrate, for the first time, that cytogenetically distinct clones in tumour tissue may also be unrelated at the molecular level.

*Received 9 October 1997*
*Revised 27 January 1998*
*Accepted 28 January 1998*

*Correspondence to:* C Jin

### MATERIALS AND METHODS

### Clinical and histopathological data

A 72-year-old woman with no prior malignant disease presented with a 3-month history of a growing tumour in the right parotid region. Cytological analysis of cells from a fine-needle aspiration biopsy was suggestive of ACC. At total parotidectomy, saving the facial nerve, a 2-cm tumour was found in the superficial lobe. The tumour was circumscribed and divided into nodules by fibrous bands. Histologically, neoplastic periodic acid-Schiff (PAS)-positive serous acinar cells were found in a microcystic pattern with moderate lymphocytic infiltrates throughout, but accentuated in the periphery. The diagnosis was low-grade malignant ACC.

### Cytogenetic techniques

The sample used for genetic analyses was taken from the excised ACC; one portion was immediately frozen at –80°C and one was used for short-term culturing and cytogenetic analysis, as described previously (Jin et al, 1995). The cell suspension obtained after mechanical and enzymatic disaggregation was subdivided into seven portions from which primary cultures L1–L7 were initiated. After 5–7 days, partial harvesting of the primary cultures was performed. The remaining cells in cultures L1–L3 were further cultured until 90% confluence (3–5 days). Then, each culture was split 1:3; one cell portion was used for DNA extraction, one was used for cytogenetic analysis and the remaining cells were plated for further culturing. This scheme was repeated until the cells spontaneously stopped dividing (passages 3–5). The cell culture morphology assessed by an inverted microscope was epithelial-like in all primary cultures and subcultures.

**Table 1** Cytogenetic findings in an acinic cell carcinoma

| Sample | Clone designation | Karyotype[a] |
|---|---|---|
| L1 | C1 | 46,X,t(X;2)(p11;p11)[3] |
| | C2 | 46,XX,der(1)t(1;6)(p32;p21),der(6)t(1;6)(p22;p21)[4] |
| | C3 | 47,XX,add(1)(p?),–2,der(3)t(2;3)(p11;q29),–17,+3mar[3] |
| | C4 | 46,XX,t(1;15)(p22;q24)[4] |
| | C5 | 46,XX,t(1;16)(p32;q13)[4] |
| | C6 | 46,XX,del(3)(p25)[3] |
| | C7 | 46,XX,t(4;5)(q25;q35)[3] |
| | C8 | 46,XX,inv(5)(q22q33)[2] |
| | C9 | 46,XX,t(2;19)(p23;q13)[3] |
| L2 | C4 | 46,XX,t(1;15)(p22;q24)[223] |
| | C6 | 46,XX,del(3)(p25)[22] |
| | C10 | 46,X,t(X;11)(q24;p15),t(1;10)(q42;q11),inv(5)(p13q11)[52] |
| | C11 | 45,X,–X,del(1)(q42),del(4)(q31),der(9)t(4;9)(q31;q32)[21] |
| | C12 | 46,XX,add(1)(q32),t(3;5)(p13;p13)[4] |
| | C13 | 46,XX,t(1;3)(p36;q12)[41] |
| | C14 | 46,XX,t(1;7)(p32;p22)[2] |
| | C15 | 46,XX,t(1;11)(p36;q13),inv(6)(p23q21)[9] |
| | C16 | 46,XX,t(1;14)(q22;q22)[4] |
| | C17 | 46,XX,del(2)(p21),der(3)inv(3)(p21q29)t(2;3)(p21;p21)[14] |
| | C18 | 46,XX,inv(2)(p25q21)[2] |
| | C19 | 46,XX,inv(3)(p25q21)[13] |
| | C20 | 46,XX,der(3)t(3;18)(q27;q21),der(4)t(4;5)(q31;q22),der(5)t(5;13)(q13;q14), der(13)add(13)(q11),der(18)t(3;18)(q27;q21),der(19)t(4;19)(q31;q13)[19] |
| | C21 | 46,XX,t(3;6)(p21;q25),t(3;13)(q13;q33),t(11;16)(q13;q22)[12] |
| | C22 | 46,XX,der(4)t(4;9)(q35;q22),der(9)t(9;10)(q22;q11),inv(10)(q11q24),del(10)(q11)[3] |
| | C23 | 46,XX,add(6)(q27)[8] |
| | C24 | 46,XX,t(7;13)(p15;q14)[17] |
| | C25 | 45,X,–X,t(10;16)(q11;q22),t(9;20)(q22;q13)[4] |
| L3 | C19 | 46,XX,inv(3)(p25q21)[2] |
| | C26 | *46,X,add(X)(q13),add(3)(q11),del(4)(q?),add(4)(q11),der(5)t(4;5)(q11;q31),del(14)(q22)[1] |
| | C27 | 46,X,t(X;5)(q28;q13),inv(12)(p13q24)[3] |
| | C28 | 46,X,t(X;8)(q23;p23),t(1;14)(p34;q32)[5] |
| | C29 | 46,XX,t(1;9)(p32;q34)[10] |
| | C30 | 46,XX,t(2;5)(q11;q35),t(9;15)(p22;q15),t(10;12)(q22;p13)[9] |
| | C31 | 46,XX,t(3;7)(p25;p22)[8] |
| | C32 | 46,XX,t(3;19)(q21;q13)[10] |
| | C33 | 46,XX,add(4)(q21),add(5)(q35),t(5;16)(q13;q24)[18] |
| | C34 | 46,XX,t(6;11)(q13;q25)[6] |
| | C35 | 46,XX,t(9;22)(p13;q11)[2] |
| | C36 | 46,XX,t(17;17)(p10;p10)[2] |
| L4 | C1 | 46,X,t(X;2)(p11;p11)[43] |
| L5 | C4 | *46,XX,t(1;15)(p22;q24)[1] |
| | C26 | 46,X,add(X)(q13),add(3)(q11),del(4)(q?),add(4)(q11),der(5)t(4;5)(q11;q31),del(14)(q22)[3] |
| | C30 | *46,XX,t(2;5)(q11;q35),t(9;15)(p22;q15),t(10;12)(q22;p13)[1] |
| | C37 | 46,XX,t(1;10)(p22;q22)[2] |
| | C38 | 46,XX,t(1;11)(p22;p15)[40] |
| | C39 | 46,XX,t(1;17)(p33;q23),t(8;14)(q22;q22)[2] |
| | C40 | 46,XX,t(3;12)(q13;p13)[21] |
| | C41 | 46,XX,add(5)(p11),t(6;11)(p21;p15),–12,del(16)(q22),+mar[8] |
| | C42 | 46,XX,t(6;19)(p21;q13)[10] |
| | C43 | 46,XX,t(12;15)(p13;q22)[8] |
| L6 | C38 | 46,XX,t(1;11)(p22;p15)[5] |
| | C39 | 46,XX,t(1;17)(p33;q23),t(8;14)(q22;q22)[5] |
| | C40 | 46,XX,t(3;12)(q13;p13)[29] |
| L7 | C33 | 46,XX,add(4)(q21),add(5)(q35),t(5;16)(q13;q24)[3] |
| | C44 | 46,X,del(X)(q24),t(1;6)(q32;q26),add(8)(p11)[7] |
| | C45 | 46,XX,t(11;17)(p11;p11)[29] |
| | C46 | 46,XX,t(14;16)(q13;q24)[2] |
| | C47 | 46,XX,t(17;22)(q21;q13)[26] |

[a]Karyotypes marked with an asterisk were present in clonal proportions in other cultures.

**Figure 1** Genetic convergence during in vitro culturing of a parotid gland ACC. p, passage

G-banding was obtained with Wright's stain. The description of the karyotypes followed the recommendations of the International System for Human Cytogenetic Nomenclature (ISCN, 1995).

### Molecular genetic analysis of X-chromosome inactivation pattern

Total DNA extraction from primary tumour tissue, peripheral blood and cell cultures was performed using standard procedures (Sambrook et al, 1989). An aliquot of 5 μg of DNA was digested with the appropriate enzymes (Abrahamson et al, 1990), blotted on to nylon filters (Genescreen, Dupont) and hybridized with $^{32}$P-labeled DXS255 (M27β) DNA (Church et al, 1984; Abrahamson et al, 1990). Hybridizations and washings were as described by Church and Gilbert (1984).

### RESULTS

### Cytogenetic findings

A total of 1164 cells from the seven primary cultures (L1–L7) was analysed. Of these, 235 (20%) had a normal chromosome complement, 109 (10%) had non-clonal aberrations, and the remaining 820 (70%) karyotypically abnormal metaphase cells gave rise to 47 cytogenetically unrelated clones altogether (Table 1). The number of cells in each clone varied from 2 to 223. Ten of the abnormal clones were found in more than one independent primary culture. The karyotypic changes were diverse, and all chromosomes, except chromosome 21, were involved. Chromosome 6 was involved in eight clones: three had rearrangements of 6p21 and five had structural rearrangements affecting 6q.

**Figure 2** Clonality analysis using the probe DXS255 and *Pst*I and *Hpa*II-double digested DNA. Lane 1, DNA from tumour biopsy digested with *Pst*I; lane 2, DNA from tumour biopsy digested with *Pst*I and *Msp*I, to determine the sizes of the unmethylated *Hpa*II-digested alleles; lane 3, DNA from tumour biopsy digested with *Pst*I and *Hpa*II; lanes 4–6, DNA from culture L1 at passage 5, culture L2 at passage 3 and culture L3 at passage 3, respectively, digested with *Pst*I and *Hpa*II. Bottom, a schematic illustration of the site recognized by DXS255 (adapted from Abrahamson et al, 1994). Variable: variable sequence causing different allele sizes; P, *Pst*I site; H*, *Hpa*II site (methylation sensitive); M, *Msp*I site (methylation insensitive)

Cell subcultures from three (L1–L3) of the seven lines were further studied cytogenetically after continued in vitro culturing. In all three lines, the cytogenetic complexity decreased with time, i.e. the initial polyclonality was reduced to near monoclonality with

© Cancer Research Campaign 1998

one clone making up 82–96% of the cells after 3–4 passages (Figure 1): C48 [46,XX,t(15;18)(p10;q10)] in L1, C21 [46,XX, t(3;6)(p21;q25),t(3;13)(q13;q33),t(11;16)(q13;q22)] in L2 and C49 [46,XX,t(1;9)(p34;q22),t(4;9)(p16;q13),t(9;13)(p22;q14)] in L3. The cell populations that took over the cultures were different in the three lines and were either very small (C21 was found in 12 of 599 cells and C49 was present only as a single cell) or could not be detected at all (C48) in the primary cultures.

### Molecular findings

The patient was shown to be heterozygous for the polymorphic marker DXS225 by analysis of DNA from peripheral blood (data not shown). To determine the X-chromosome inactivation status, DNA extracted from lines L1–L3 and frozen tumour tissue was digested with *Pst* I, giving rise to the polymorphic restriction fragments, and the methylation-sensitive enzyme *Hpa* II. Analysis of the primary tumour tissue showed one dominating clone with respect to X-chromosome inactivation, with one allele being partially and one completely methylated, whereas cell cultures L1 and L3, each consisting of one dominating cytogenetic clone (Figure 1), showed opposite X-inactivation patterns, one of the two alleles being completely methylated and the other completely unmethylated (Figure 2, lanes 4 and 6). The remaining culture, L2, showed the same methylation pattern as the tumour tissue. All analyses of DNA from cell cultures and tumour tissue were repeated with identical results.

### DISCUSSION

ACC is an uncommon, usually low-grade, malignancy of the salivary glands. Including the present case, only 11 ACCs with abnormal karyotypes have been reported (Mitelman, 1998). All of them have displayed pseudo- or near-diploid chromosome numbers with simple karyotypic changes and, excluding clones with sex chromosome aberrations as the sole anomaly, four of them have had cytogenetically unrelated clones. The only recurrent changes that have been identified are all numerical, i.e. – Y (six cases), + 8 (three cases) and + 7 (two cases). It may also be noted that four of the ACCs had structural rearrangements of 6q, a chromosome arm frequently involved in other types of salivary gland carcinoma (Mitelman, 1998).

The ACC of the present study was highly polyclonal at cytogenetic analysis of the primary cultures. By obtaining relatively pure subcultures, each containing one dominant abnormal clone, the cell cultures became suitable for analysis of their X-chromosome inactivation patterns. In women, one of the two parental X-chromosomes is randomly inactivated in each somatic cell in early fetal life; the same pattern of inactivation is then stably transmitted to the daughter cells at every cell division. It follows, then, that the finding of completely opposite methylation patterns in cultures L1 and L3, each of which had a single abnormal clone (C48 and C49 respectively) making up 96% of the total number of cells, must indicate that the two dominant clones had originated from different cells. The cleavage patterns observed for DNA from primary tumour tissue and culture L2 (Figure 2, lanes 3 and 5) most probably resulted from the combination of sampling from less homogeneous cell populations (the dominating clone in L2 constituted only 82% of the cells) and intercellular variation in the degree of methylation at the CCGG site, recognized by the *Hpa* II enzyme, on inactive X chromosomes (Hendriks et al, 1992).

All the abnormal clones in the ACC, both in primary cultures and after in vitro passaging, had relatively simple karyotypes, and it may be argued that only a few, or even none, of them were representative of the tumour parenchyma. We cannot entirely dismiss this possibility, but, as outlined above, all previously reported cytogenetically abnormal ACCs have had relatively simple, near-diploid karyotypes and have often been polyclonal. The finding of near-diploid clones in the present case is also in agreement with DNA flow cytometry data on a series of 15 ACCs, showing that low malignant cases have a diploid DNA content (el-Naggar et al, 1990). Finally, it should also be emphasized that the patient had not received any genotoxic treatment and that, although cytogenetically abnormal clones may be found in non-neoplastic short-term cultured mucosa samples, these cultures have never displayed as many aberrations as the present ACC (Jin et al, 1997*b*).

### REFERENCES

Abrahamson G, Fraser NJ, Boyd Y, Craig I and Wainscoat JS (1990) A highly informative X-chromosome probe, M27β, can be used for the determination of tumor clonality. *Br J Haematol* **74**: 371–377

Church GM and Gilbert W (1984) Genomic sequencing. *Proc Natl Acad Sci USA* **81**: 1991–1996

el-Naggar AK, Batsakis JG, Luna MA, Mclemore D and Byers RM (1990) DNA flow cytometry of acinic cell carcinomas of major salivary glands. *J Laryngol Otol* **104**: 410–416

Heim S, Teixeira MR, Dietrich CU and Pandis N (1997) Cytogenetic polyclonality in tumors of the breast. *Cancer Genet Cytogenet* **95**: 16–19

Hendriks RW, Hinds H, Chen Z-Y and Craig IW (1992) The hypervariable DXS225 locus contains a LINE-1 repetitive element with a CpG island that is extensively methylated only on the active X chromosome. *Genomics* **14**: 598–603

ISCN (1995): *An International System for Human Cytogenetic Nomenclature*. Mitelman F (ed.). S. Karger: Basle

Jin Y, Mertens F, Jin C, Åkervall J, Wennerberg J, Gorunova L, Mandahl N, Heim S and Mitelman F (1995) Nonrandom chromosome abnormalities in short-term cultured primary squamous cell carcinomas of the head and neck. *Cancer Res* **55**: 3204–3210

Jin Y, Mertens F, Persson B, Gullestad HP, Jin C, Warloe T, Salemark L, Jonsson N, Risber B, Mandahl N, Mitelman F and Heim S (1997*a*) The reciprocal translocation t(9;16)(q22;p13) is a primary chromosome abnormality in basal cell carcinoma. *Cancer Res* **57**: 404–406

Jin C, Jin Y, Wennerberg J, Åkervall J, Grenthe B, Mandahl N, Heim S, Mitelman F and Mertens F (1997*b*) Clonal chromosome aberrations accumulate with age in upper aerodigestive tract mucosa. *Mutat Res* **374**: 63–72

Mitelman F (1998) *Catalog of Chromosome Aberrations in Cancer*. 6th edn. Wiley-Liss: New York

Noguchi S, Motomura K, Inaji H, Imaoka S and Koyama H (1993) Clonal analysis of fibroadenoma and phyllodes tumor of the breast by means of polymerase chain reaction. *Cancer Res* **53**: 4071–4074

Novelli MR, Williamson JA, Tomlinson IPM, Elia G, Hodgson SV, Talbot IC, Bodmer WF and Wright NA (1996) Polyclonal origin of colonic adenomas in an XO/XY patient with FAP. *Science* **272**: 1187–1190

Sambrook J, Fritsch EF and Maniatis T (1989) *Molecular Cloning: a Laboratory Manual*. Cold Spring Harbor Laboratory Press: Cold Spring Harbor, NY

Wainscoat JS and Fey MF (1990) Assessment of clonality in human tumors: a review. *Cancer Res* **50**: 1355–1360

© Cancer Research Campaign 1998